# Experimental Investigation on the Potential Use of Magnetic Water as a Water Reducing Agent in High Strength Concrete

**DOI:** 10.3390/ma15155219

**Published:** 2022-07-28

**Authors:** Malathy Ramalingam, Karuppasamy Narayanan, Jagan Sivamani, Parthiban Kathirvel, Gunasekaran Murali, Nikolai Ivanovich Vatin

**Affiliations:** 1 Department of Civil Engineering, Sona College of Technology, Salem 636 005, India; malathycivil@sonatech.ac.in (M.R.); jagan.civil@sonatech.ac.in (J.S.); 2 School of Civil Engineering, SASTRA Deemed University, Thanjavur 613 401, India; 3 Peter the Great St. Petersburg Polytechnic University, 195251 Saint Petersburg, Russia; vatin@mail.ru; 4 Division of Research & Innovation, Uttaranchal University, Dehradun 248 007, India

**Keywords:** magnetic water, workability, compressive strength, super plasticizer, SEM-EDAX

## Abstract

High-strength concrete is designed for a self-weight reduction structure and exhibits higher resistance to compressive loads. This paper proposes a novel technique to enhance concrete’s properties using Magnetic Field Treated Water (MFTW), describing the results of experimental studies to apprehend the fresh, hardened and microstructural behavior of concrete prepared with Magnetic Water (MW) using a permanent magnet with a field intensity of 0.9 Tesla. The novel scheme focuses on utilizing MW as a water-reducing agent instead of SP to improve the workability of fresh concrete with a 0.38 w/c ratio for achieving M40 grade concrete. Results show a 12% improvement in compressive strength and an 8.9% improvement in split tensile strength compared to normal water (NW) with 1% SP. At 30% cement volume reduction, Magnetic Water Concrete (MWC) performs better than Normal Water Concrete (NWC). Microstructure examination shows that a smaller Calcium Hydrate (CH) crystal is formed with MW and its mineral composition is observed through Energy Dispersive X-ray Analysis (EDAX).

## 1. Introduction

Nowadays, most metropolitan cities in India have domestic water shortage problems due to the lack of monsoon rains and depleted groundwater tables. Currently, the construction industry uses an enormous quantity of potable water for several purposes. Of especial concern in this paper, nearly 150–200 L of fresh water are consumed to produce 1 m^3^ of concrete. Water quality and quantity play a crucial part in the strength attainment of fresh and hardened properties of concrete. Concrete technologists are becoming more interested in Magnetic Water’s (MW’s) potential use in mixing concrete to enhance the concrete’s quality. In MW, water is passed through the magnetic field to become magnetized. As a result, the negative ionic hydration is increased, and potentially harmful water crystal structure is found [1]. When water is mixed with concrete, a hydration process occurs, where hydration products are developed on the facade of the cement particles, which prevent cement particles from further hydration. Adding MW allows water molecules to seep into the cement particles and enables completion of the hydration process. Therefore, the properties of concrete are improved [2]. A decrease in water molecules from 13 to 5 or 13 to 6 due to magnetic field exposure makes them participate more in the cement hydration process [3,4]. It has been found that a higher degree of hydration of cement is achieved with MW than that of normal water (NW) and the strength of concrete is compressively increased, i.e., 10–23%. As amplification of the mechanical strength depends on the intensity of the magnet, the strength can be increased with 0.8 or 1.2 Tesla (T) [5]. Mixing MFTW in concrete leads to attaining strength at the early age of 7 days [6]. The influence of a magnetic field on the water can be understood through hydrogen bonding and changes in ion charges in water. MW quality to upgrade concrete properties depends on the magnetic intensity, magnetic field exposure time and quantity of water exposed to the magnetic field [7]. In excess of a trillion metric tons of water are used in India to produce concrete [8]. It is essential to satisfy the quality parameters of water for mixing and curing, which helps achieve the required concrete performance. The scarcity of potable drinking water makes such magnitudes of water utilisation a major issue, and the usage of MW in concrete can decrease the consumption of water and can improve the quality of NW [9]. In addition, the MW improves the workability, strength and durability of concrete [10].

Research has mainly focused on reducing water admixtures with super plasticizers (SPs) to achieve high workability while producing high-strength concrete [11]. An increase in water to cement ratio improves concrete’s workability, but the compressive strength is decreased. Workability and strength can be improved when concrete is mixed with MW [12]. When preparing the fresh concrete, 0.71 m/s water current velocity and 4.5 s/L time treatment are ideal [13]. The MW treatment plays an important role in controlling scale formation. By changing the magnetic field intensity, the hardness of water is reduced by 51%. When water is passed at high speed through the magnetic field, the hardness of water is reduced. The MW treatment has the main advantage of increasing concrete efficiency and being eco-friendly. The MW processes are helpful to reduce time, energy, water, chemical cost, protection against corrosion, etc. [14]. Concrete specimens are cast without MW as a reference and others with MW of w/c 0.45, 0.5 and 0.55. The water structure is oriented in one direction, increasing hydration, and the bond angle changes. It has been found that the slump value is increased by about 25% for a w/c ratio 0.55 with MW, and the weight of the concrete specimen is decreased by 3%. The strength is improved by 10%, when using MW with the reduction of cement up to 75% [15]. The performance of concrete with MW treated with the different magnetic intensities of 0.8 T, 0.9 T and 1 T has been is analyzed. It has been shown that the MW contributes positively to the compressive strength of concrete.

The porosity and water absorption sorptivity are higher in MW passing through the magnetic intensity of 0.8 T and 0.9 T, but less with 1 T [16]. Measurements of the water’s physical parameters, such as its specific heat and boiling point, are made using NW and four different kinds of MW. Magnetizing equipment has been developed with 26 magnets with a minimum strength of 280 millitesla (mT). There are four samples: MW-1 (100 mT), MW-2 (200 mT), MW-3 (300 mT) and MW-4 (400 mT). The evaporation rate of MW is high. When comparing with NW, the boiling point is lower for MW and the optimal magnetization effect is 300 mT [17]. Without comprising the strength parameter, the impact of MW over NW while mixing concrete leads to reducing the water content by 10% and the SP dosage by 30% [18]. Low impact on the environment and reasonable cost is attained through the usage of Magnetic Field Treated Water (MFTW) for concrete mixing [19]. Through Scanning Electron Microscopy (SEM) and environmental analysis, it has been proved that the suspended particulates in MW start to split and re-arrange, resulting in less vibration during compaction time and zero air cavities present in magnetic concrete. The increases in water particle numbers reduces their size by 51% because of the magnetic field effect on water [20]. Recent research articles focus on improving the performance of special concrete, e.g., fiber reinforced concrete and SCC using magnetized water [21,22,23]. Producing cost-effective and high-strength concrete with locally available water reduces the water-cement ratio. Another challenging issue is achieving the workability of high-strength concrete with a low water-cement ratio. It is observed from previous research articles, that granite poweder (GP) waste can be added as a replacement for cement at 10 to 40% dosages. The research proved the usefulness of GP waste as an alternative for cement in concrete mix, bringing to light the overall reduction in cement consumption leading to sustainability and eco-friendliness [24]. Safiuddin et al. (2007) investigated the gel-space ratio and degree of hydration in concrete produced with the addition of GP waste. It was found that in concrete mixes containing 20 to 40% GP waste, more than 40% of the GP waste remained passive even with curing at 28 days. Even so, the hydration of cement in granite powder waste added concrete mix was enhanced owing to relatively high water-cement ratio in the concrete paste [25]. Some researchers reported that concrete mix containing a large volume of granite powder showed a relatively low level of reaction by the GP as compared to conventional concrete mix. It was found that after a 90-day curing period, 20% of the GP in the concrete mix remained non-reactive. On the other hand, appropriate water-cement ratios enhanced the hydration of cement in concrete mixes prepared with a large quantity of GP. Furthermore, it was noted that the gel-space ratio was in better agreement with compressive strength [26]

This paper focuses on studying high-strength concrete’s fresh and hardened properties using MW as a water-reducing agent instead of SP. Here, the water is magnetized through PERMAG (N407) with a field intensity of 0.9 T, and the fresh concrete specimens were prepared by varying SP dosage (from 0 to 1% by volume of cement). Moreover, the effect of MW with different exposure times and storage time on fresh concrete properties is optimized. In addition, granite wastage was also examined as a partial replacement for cement volume with different substitution levels mixed with MW. This research also included microstructural and energy dispersion studies on concrete samples to evaluate the hydration rate of C-S-H crystal and element identification while mixed with NW and MW. The main contributions of this paper are to examine the effect of fresh and hardened concrete properties of high-strength concrete using MW without the addition of SP, and to incorporate granite waste as a filler material for cement with an equal interval of addition mixed with MW. The proposed approach will help improve water quality in the concrete industry due to the water magnetization, which directly enhances the quality and durability of the structures.

## 2. Experimental Program

### 2.1. Materials and Mix Proportioning

Portland cement (Type I) has been used in this study, with a specific density of 3.10 g/cm^3^. This cement has many physical properties, such as fineness, initial and final setting time, consistency, specific gravity and chemical composition, and the test values are shown in Table 1 and Table 2, respectively.

**Figure 1:**
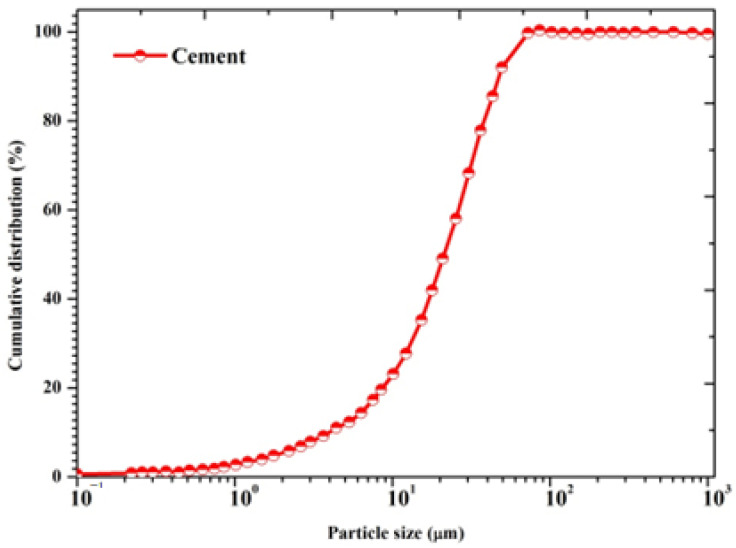
Particle size distribution of OPC.

The specific surface area of cement is 3268 cm^2^/gm and the particle size distribution of cement [28] is displayed in Figure 1. 

The physical properties of fine and coarse aggregate with 20 mm sizes are depicted in Table 3 as per IS: 383-1970 [29]. The MW has been prepared with a permanent magnet (0.9 T) of PERMAG N406. Due to magnetization, water particles get charged and the molecules inside the water cluster decrease from 13 to 5 or 6, which eventually decreases the hardness of water thus improving the strength of concrete when compared to use of normal water (NW).

The chemical parameters of normal water after magnetization get changed and its quality is improved [3]. The chemical parameters of NW and MW are shown in Table 4 as per IS: 10500-2012 specification [30]. The mix design for M40 grade concrete has been carried out as per IS: 10262:2009 [31] and with reference to IS: 456:2000 [32]. The water-cement ratio has been taken as 0.38. 

Table 5 represents the mix proportions for 1 m^3^ of concrete. In our research work we have casted 87 specimens in total. To study fresh concrete properties we have casted 30 different concrete mixes (6 concrete mixes with different SP dosage, six concrete mixes with different exposure time, 6 concrete mixes with retention time, 12 concrete mixes with different storage time), while for hardened concrete properties we casted 57 concrete specimens.

The granite waste used as a replacement for cement volume is obtained from the locally available granite processing industry in Salem, India. The specific gravity and fineness of granite waste are about 2.58 and 2.61 m^2^/g, respectively, which is the same as the fineness of the cement. Characterization of granite waste was performed using an X-ray Diffraction (XRD) technique.

### 2.2. Experimental Methodology

**Figure 2:**
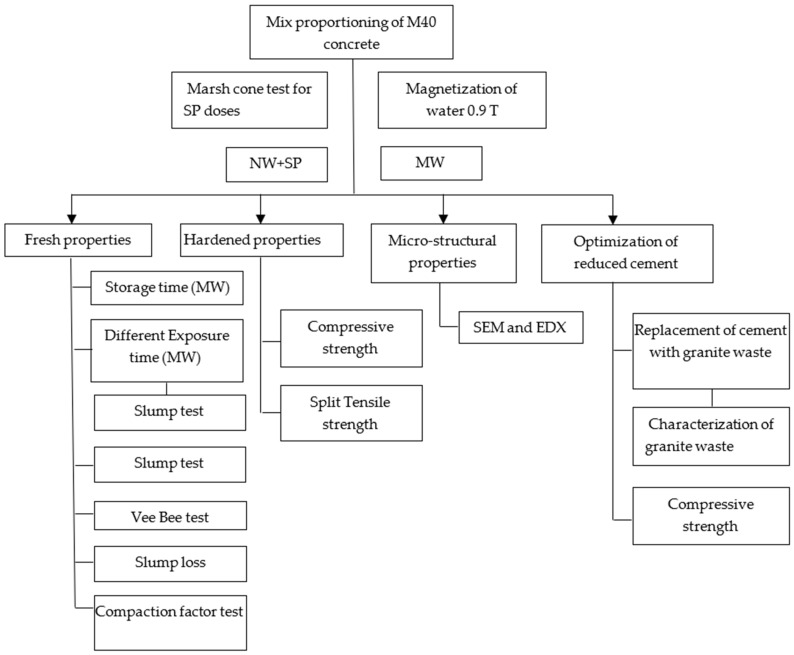
Study framework.

The experiment starts with a mixed proportion of M40 grade concrete with a water-cement ratio of 0.38, shown in Figure 2.

To obtain the desired workability, the SP has been used as water reducing admixture and its dosage has been optimized by conducting the Marsh cone test. Then, the performance was evaluated through a slump cone test and slump resting time (NW with optimized 1% SP and MW without SP) by varying magnetic exposure time from 0 to 60 min with 15 min intervals. Fresh properties of M40 grade concrete were measured by conducting three tests: slump cone, Vee-bee and compaction factor (NW with optimized 1% SP and MW without SP) at different storage periods, i.e., 0, 15 and 30 min. Hardened concrete properties, such as compressive strength at 3, 7, 14, 21 and 28 days and split tensile strength, were measured at 7, 14 and 28 days of concrete from casting. The concrete cubes were prepared with MW by replacing cement volume by 30% with granite powder. The mineralogical studies of the granite waste were performed using XRD. Microstructure characterization of concrete samples (NW with optimized SP and MW without SP) were examined after 28 days of the curing period through SEM and EDAX.

### 2.3. Marsh Cone Test

The Marsh cone test was conducted to optimize the SP dosage to achieve cement’s flow ability with a 0.38 water-cement ratio and varying percentage of SP from 0 to 1.4% with an increment of 0.2%. The saturation point was obtained by observing the time taken to collect 500 mL of cement paste passing from the nozzle of the Marsh cone. The test was conducted as per ASTM D6910 [33].

### 2.4. Fresh Concrete Properties

#### 2.4.1. Slump Test

The slump cone test is a standard method used to evaluate concrete mix consistency as per IS: 1199:1959 (2004) [34]. The workability test was carried out using NW and MW by varying SP (Conplast SP430) up to 1% with an increasing rate of 0.2%. Three layers of concrete mix were made and poured into the cone; the tamping rod was used to compact each layer 25 times. When the top layer was rounded, the mold was immediately separated from the fresh concrete by lifting gradually in the vertical direction. Then, the slump value of MW was noted by varying the magnetic field exposure time, i.e., 0, 15, 30, 45 and 60 min.

#### 2.4.2. Slump Loss

The fresh concrete may lose its workability with respect to time, known as slump loss. The retention time of slump depends on the factors like types and combinations of concrete materials used, ambient conditions, and the total quantity of utilized water [35]. The slump loss test was carried out with a 0.38 w/c ratio using NW with optimized 1% SP and MW without SP. Immediately after preparing the concrete mix, the concrete was transferred to a wheelbarrow and the slump test was performed. The slump loss was measured at 5 min intervals for a maximum duration of 30 min. Before each slump measurement, the concrete was mixed manually. The concrete was kept covered in the wheelbarrow until the slump loss study was over.

#### 2.4.3. Compaction Factor Test

The compaction factor test was performed for concrete, which has low workability. The setup consists of an upper, lower hopper and cylinder. The test was performed as per IS: 1199: 1959 [31]. The concrete mix was prepared (NW with optimized 1% SP and MW without SP) after magnetization under different storage periods, i.e., 15 and 30 min.

#### 2.4.4. Vee Bee Test

Vee bee is used to find the consistency and mobility of recently mixed concrete using a Vee-bee consistometer, and it was performed as per IS: 1199:1959 (2004) [34]. The sample of fresh concrete mix was prepared (NW with optimized 1% SP and MW without SP) with different storage times after magnetization. This test was used to measure the relative effort required to change a mass of prepared fresh concrete sample from one definite shape to another (i.e., from conical to cylindrical) in terms of vibration.

### 2.5. Hardened Concrete Properties

#### 2.5.1. Compressive Strength Test

The compressive strength of concrete cubes has been evaluated as per IS: 516:1959 [36]. Two sets consisting of 15 numbers with 150 × 150 × 150 mm size were cast (NW with optimized 1% SP and MW without SP), and after 28 days, they were tested under a compression testing machine of 100 T capacity (FIE). The load was applied with a pace of 0.05 MPa/s and the specimen was tested for ages 3, 7, 14, 21 and 28.

#### 2.5.2. Split Tensile Test

The cylindrical specimen was prepared with 150 mm diameter and 300 mm height, and the NWC and MWC concrete were tested for tensile strength as per 516:1959 [36]. After 3, 7, 14, 21 and 28 days of curing, the test was performed by inserting a cylinder-shaped specimen in a horizontal position between the loading surfaces.

### 2.6. Micro Structural Studies

Concrete is highly heterogeneous and has a complex microstructure, and it consists of three components, i.e., hydrated cement paste, aggregate and interfacial transitions zone between the cement pastes and aggregate. The NWC and MWC samples of M40 grade concrete were characterized by SEM of model XL30 equipped with an EDAX spectrometer for X-ray dispersion element analysis, and micrographs images were taken from the surface of cement paste specimen [37].

## 3. Discussion of Results

### 3.1. Characterization of Granite Powder 

**Figure 3:**
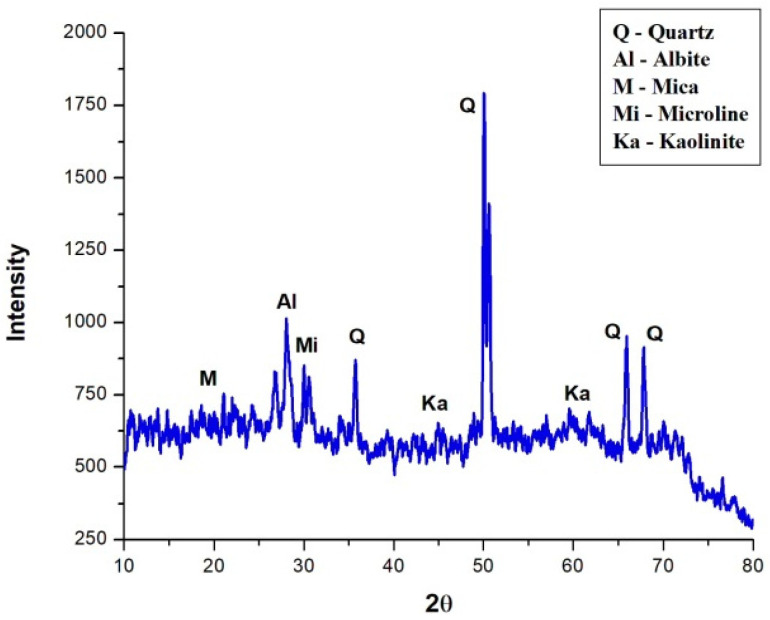
X-ray diffraction of granite powder.

The chemical composition of of the granite powder waste is about 73% Silica (${\mathrm{SiO}}_{2})$, 12% Alumina (${\mathrm{Al}}_{2}O_{3}$), 0.5% Magnesia (MgO), 3.7% Potassium Oxide $\left(K_{2}O\right)$, 1% lime and 1.9% iron; these properties are suited for producing concrete [38]. The mineralogical study of the granite waste was performed using X-ray diffraction technique using a D/tex Ultra 250 1D detector, with power diffraction by a factor of 250 in speed and 20% energy resolution. The result from the mineralogical classification in terms of XRD of the granite waste is depicted in Figure 3.

It has been observed that granite waste contains more intense peaks of Quartz (PCPDF no.: 89-8935), Albite $\left({\mathrm{NaAlSiO}}_{3}O_{8}\right)$ (PCPDF no.: 89-6430), Microline $\left({\mathrm{KalSi}}_{3}O_{8}\right)$ (PCPDF no.: 89-1789), Kaolinite $({\mathrm{Al}}_{2}{\mathrm{Si}}_{2}O_{5}{\left(\mathrm{OH}\right)}_{4}$ (PCPDF no.: 75-1593) and the less intense peak of Mica (PCPDF no.: 02-0227). From the chemical and mineralogical analysis, it is observed that the presence of ${\mathrm{SiO}}_{2}$, ${\mathrm{Al}}_{2}O_{3}$ and $P_{2}O_{5}$ in the granite waste is due to quartz compounds, the main compounds present in the granite rock [39]. The concrete paste comprising high volumes of granite powder has a relatively low level of granite powder reaction compared to the conventional concrete mix [26]. Even though granite waste has already been in vogue as a partial replacement material for cement, few works have assessed the degree of hydration of granite waste added to concrete up to 40% (Safiuddin et al., 2007) [25]. Since no reactive components are present in the granite powder, this can be used to fill the volume of the cement by up to 30%.

### 3.2. Marsh Cone Test

**Figure 4:**
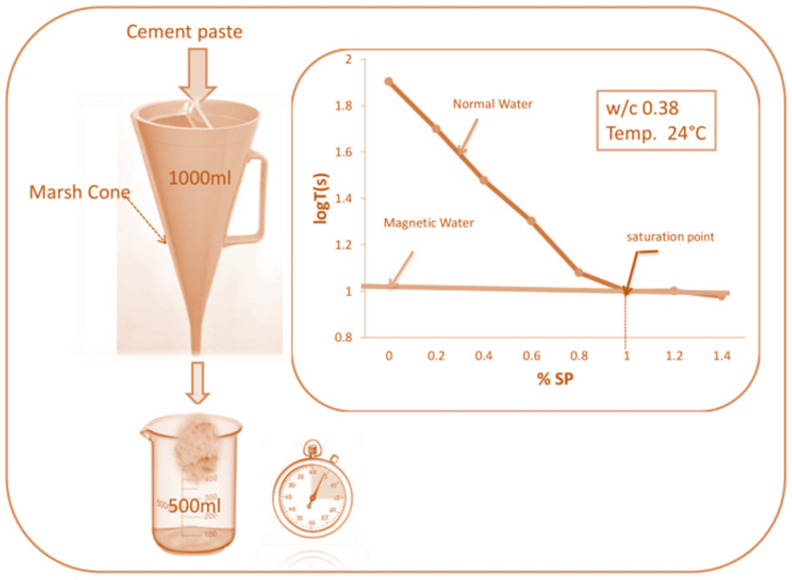
Marsh cone test results.

The Marsh cone test was conducted to optimize the uses of SP and compatibility between cement and SP to attain the required flow ability of concrete. The time taken to collect 500 mL cement paste was measured. Figure 4 shows a curve plotted between the percentage of plasticizer and log time in seconds.

It can be observed from Figure 4, that the saturation point is 1% SP in NW and MW flow ability is achieved without SP. This is because the MW changes the absorption capability of the liquid-gas interface, which tends to increase the admixture’s solubility and dispersion effect. Thus, the MWC requires zero amount of SP dosage to spread water molecules in the concrete mix to impart strength [40].

### 3.3. Fresh Concrete Properties 

#### 3.3.1. Slump Test

**Figure 5:**
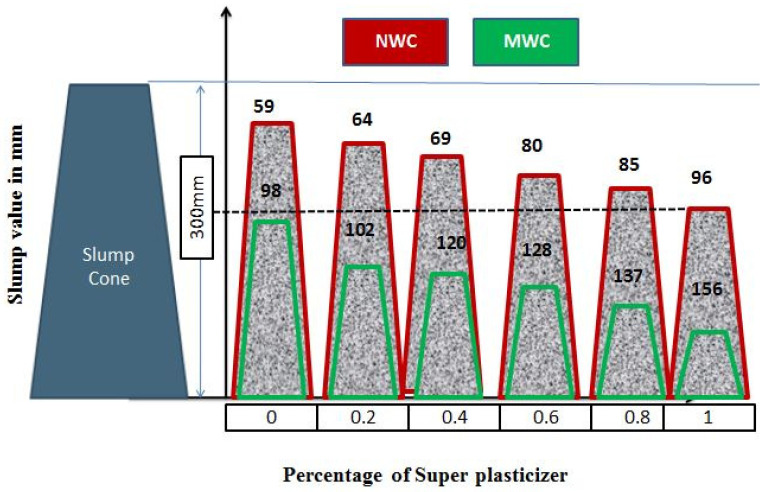
Slump values of NW and MW with 1% of SP.

The slump flow test is a frequently used method to evaluate the fresh concrete mix workability. Figure 5 illustrates the effect of SP dosage on slump values with the mixture of NW and MW in concrete. The slump value of NW with 1% SP (96 mm) was achieved in MW without SP (98 mm).

**Figure 6:**
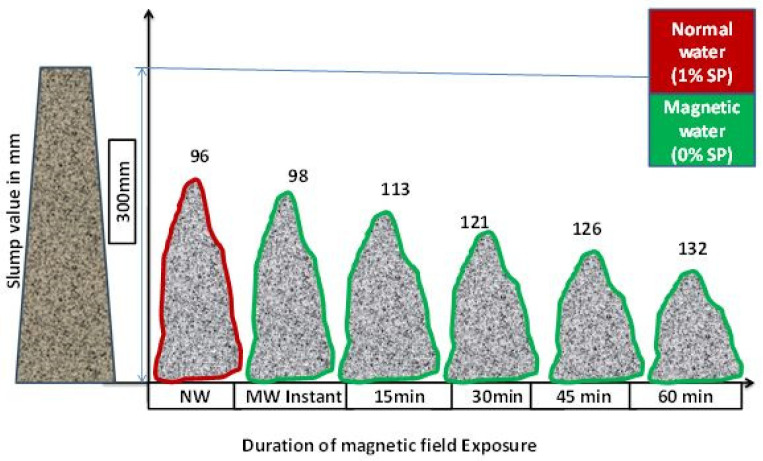
Different exposure time is slump values of concrete (NW with 1% optimized SP and MW with 0% SP).

Due to magnetization, the field produces a dispersion effect on water clusters due to magnetization and separates them into minor groups. Consequently, water molecules can readily pass through cement particles, where they can react with other cement particles. Thus, the magnetic field enhances the role of water in concrete, such as cement hydration and better flow ability [18]. Hence, the MWC does not need a water-reducing admixture. The MW itself can be a water-reducing agent for improving workability. Figure 6 shows the slump value of concrete prepared with the MW in different magnetic field exposure times up to 1 h with 15 min intervals.

It can be noticed from Figure 6 that the workability of fresh concrete mix prepared with MW without SP was increased up to 37% compared to the NW slump with an optimized 1% SP. The MW had a higher impact with respect to the exposure time when compared to instant magnetization, as shown in Figure 6. The slump value of MW with 60 min exposure was 132 mm, whereas for instant MW it was 98 mm, increasing with an increase in magnetic field exposure time. When water is exposed to a magnetic field, it is takes time to orient into the new form of MW [41]. The increases in slump achieved with magnetic exposure time have a better spreading effect and the water’s surface area is increased. When cement comes into contact with MW, more cement particles are hydrated and the strength parameters are improved [42]. As the slump value of the MWC with instant magnetic field exposure achieves the desired workability the same as that of NWC with 1% SP, further experiments have been executed with instant MW.

#### 3.3.2. Effect of Resting Time on Slump Loss

**Figure 7:**
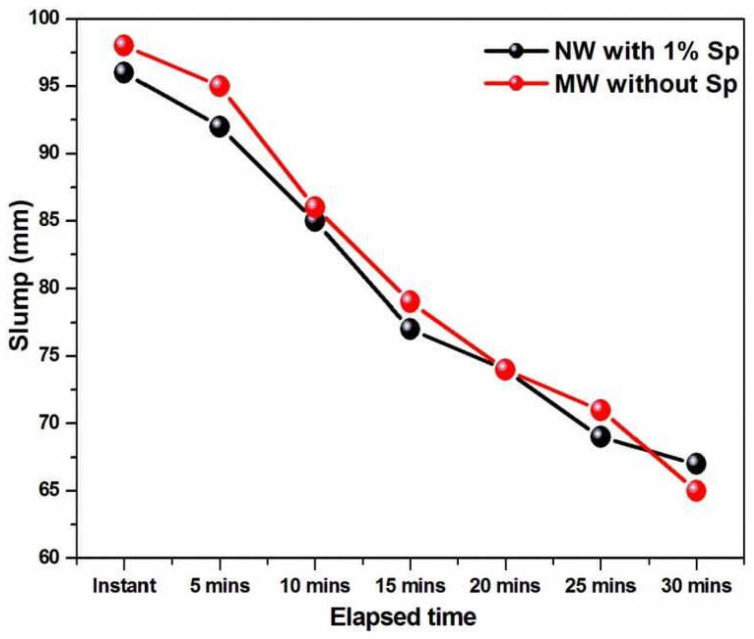
Slump loss of concrete (NW with 1% optimized SP and MW with 0% SP).

Slump loss in fresh concrete is distinct from the loss of consistency in the concrete mix with respect to resting time. Figure 7 illustrates the slump loss of the concrete mix produced with NW (with optimized 1% SP) and MW (without SP) for different resting periods.

The results indicate that the slump loss of concrete prepared with MWC with respect to retention time indicates a similar trend to that of the NWC made with 1% SP. This implies that when long-standing resting is involved, MW acts as a super lubricant and impacts the electrical load of the cement particles that cause the separation of charges from each other. Thus, the MW easily penetrates the core region of cement particles and facilitates better lubrication for a longer resting duration [43]. Hence, it is noted that concrete with both (NW with optimized 1% SP and MW without SP) should be used within 20 min of resting to achieve minimum slump. It was observed that the slump loss due to the MW was the same as the SP.

#### 3.3.3. Effect of Magnetic Water Storage Period on Its Fresh Concrete Property

##### 3.3.3.1. Slump Cone Test

**Figure 8:**
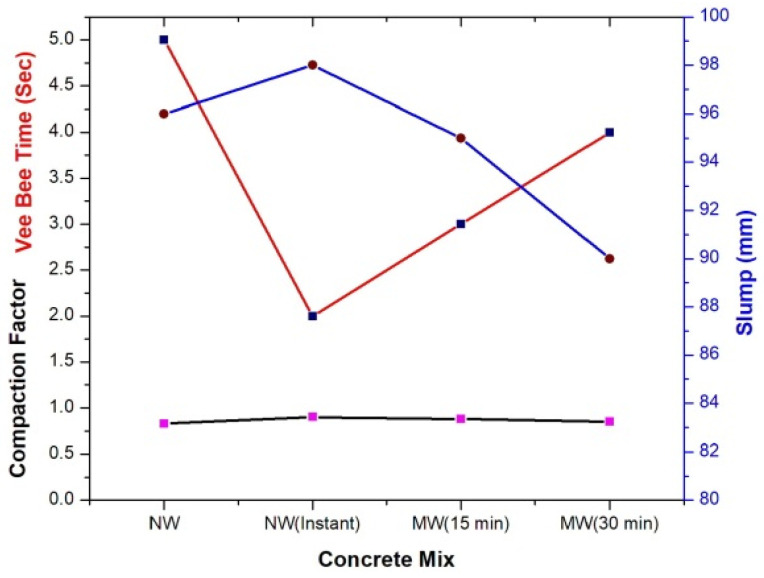
Fresh concrete properties (NW with 1% optimized SP and MW with 0% SP).

In Figure 8, the result of the slump value is shown with the NW and MW mixed at 0, 15 and 30 min storage time after magnetization. It can be noted from the results that the stored MW loses its magnetic property with respect to time, resulting in a decrease in the workability of fresh concrete.

The slump value is increased due to electrically charged water molecules due to magnetization, which improves the water interaction among cement contents on fresh concrete. It can also be noted that the magnetization effect is reduced with storage time once it leaves the magnetic field [5].

##### 3.3.3.2. Compaction Factor Test

The compacting factor test was performed to measure the workability of concrete when the slump value is less than 50 mm. The compaction factor of MWC without SP increased by 5.8% compared to NWC with optimized 1% SP. There is no significant effect on the compaction factor of concrete at 15 and 30 min after magnetization. This phenomenon is because the MW needs only a lower volume of water surrounding the cement particles to activate the hydration process without segregation and bleeding on concrete [44].

##### 3.3.3.3. Vee-Bee Test

Figure 8 represents the Vee-bee time of concrete mix prepared (NW with optimized 1% SP and MW without SP) at 0, 15 and 30 min storage time after magnetization. The experimental results show that the Vee-bee time was increased with an increase in the storage period of the MW because due to the memory possessed by water, water tries to behave in its previous form of non-MW [26]. The Vee-bee test value is optimum at a 0 min storage period with an increase in the slump compared to the NW. At instant storage time, the MW acts as a super lubricant, which penetrates easily into cement particles to facilitate better workability in a quick time [3].

### 3.4. Hardened Concrete Properties

#### 3.4.1. Compressive Strength Test

**Figure 9:**
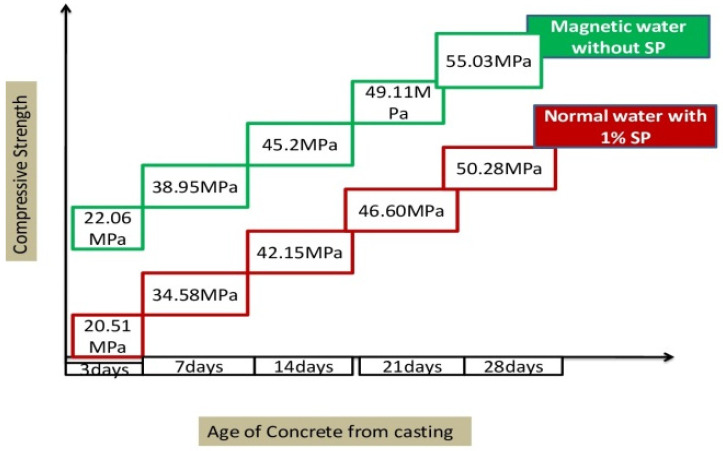
Compressive strength of concrete (NW with 1% optimized SP vs. MW with 0% SP).

Figure 9 demonstrates the compressive strength of concrete due to the effect of NW incorporated with optimized 1% SP and MW incorporated without SP. Magnetization of water with zero substitution of SP in concrete shows improvement in compressive strength by 12% at 28 days (55.03 MPa). The MFTW of some electrical charges surrounds the cement particles. These particles repel each other and form smaller water clusters, penetrating the hydration layer without difficulty and enhancing the hydration process [5].

**Figure 10:**
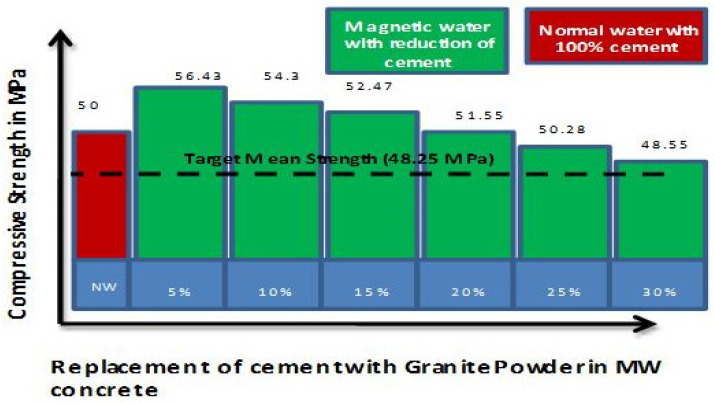
Compressive strength of concrete with cement reduction (NW with 1% optimized SP vs. MW with 0% SP).

Figure 9 shows that the target mean strength was achieved at 21 days (49.11 MPa), when MW is used in concrete. This implies that dispersion or higher spreading enable the MW particles to react entirely with cement and, thus, increases mechanical properties [15,45,46]. Figure 10 shows the target mean strength of concrete cube prepared with MW without SP attained by reducing the cement content up to 30% with granite waste.

**Figure 11:**
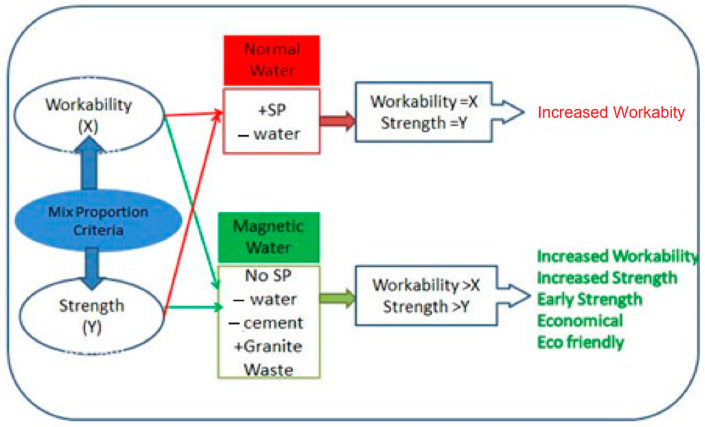
Function of MW in concrete.

Figure 11 depicts that the concrete with MW improved workability and attains early strength with a 30% reduction of cement volume. As there is a reduction in cement and no SP, this concrete is cost-effective and granite waste has been utilized, making the concrete eco-friendly. Adding the MW and granite powder in concrete enhances the hydration process and the particle packing, which directly impacts the mechanical properties. Moreover, the MW acts as a super lubricant to form a more homogeneous mixture to make complete hydration of cement.

The usage of MW in concrete enhances its strength and improves the quality of the NW. Using magnetic water instead of normal water helps to produce sustainable concrete with reduced cement content and clean compared to the methods above, such as incorporating nanomaterials or chemical admixtures [47].

#### 3.4.2. Split Tensile Test

**Figure 12:**
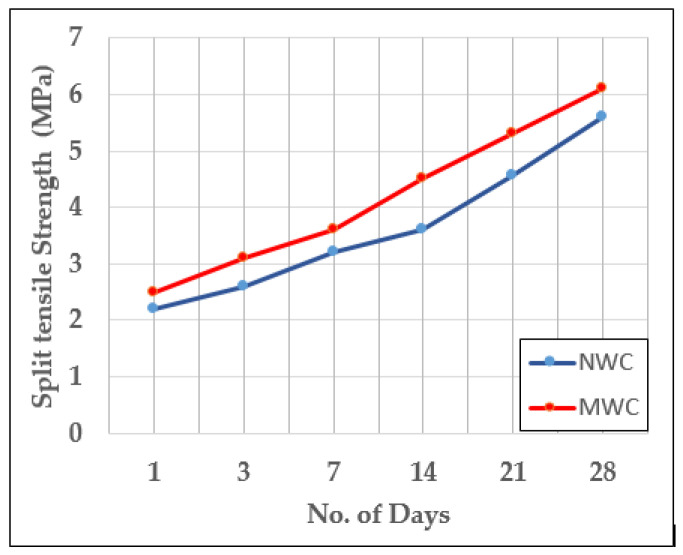
Split tensile strength of concrete (NW with 1% optimized SP Vs MW with 0% SP).

The split tensile strength of the concrete specimen prepared (NW with optimized 1% SP and MW without SP) and cast after 7, 14 and 28 days, is illustrated in Figure 12.

Similar to the strength improvement in compressive test results, the split tensile strength of concrete specimens with the MW, irrespective of all ages was higher than that of the specimens prepared with NW. The 28th day split tensile strength values for the specimen with the MW were increased by 8.9%.

### 3.5. Micro Structural Studies

**Figure 13:**
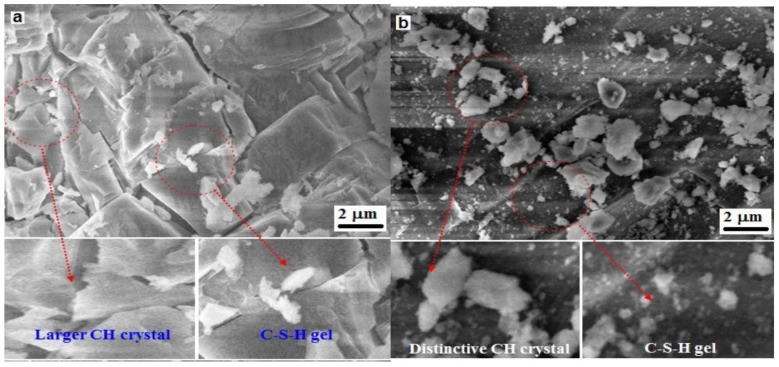
SEM image of concrete powder samples (**a**) NW with 1% optimized of SP (**b**) MW with 0% SP.

The concrete sample cast with the NW consisting of 1% SP and the MW consisting of 0% SP was tested for SEM analysis for the instant duration. Figure 13a shows the SEM image of NWC in which CH crystals exist in a larger volume in the concrete prepared with the NW. The NW tends to agglomerate the water molecules and form clusters. After the reaction of cement with water, the CH plates are produced and packed in the transition zone [6]. Figure 13b shows the SEM image of the MWC formed by adding magnetized water in concrete, which in turn reduces the size of CH crystals; the results are separated in the proper existence of C-S-H gel formation [48]. The morphology of CSH gel becomes thicker and less porous as the MW increases, which increases the mechanical strength [37].

**Figure 14:**
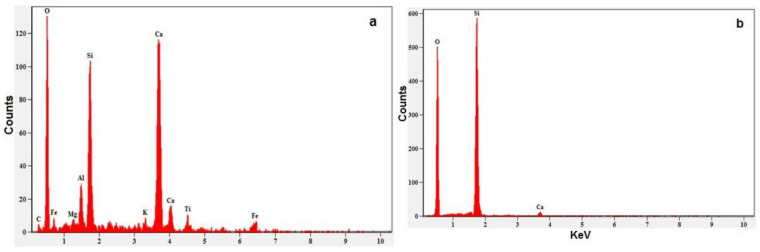
EDAX of concrete powder sample (**a**) NW with 1% optimized SP (**b**) MW with 0% SP.

Water clusters may be broken up into single molecules or smaller clusters by the process that incorporates magnetic force in the magnetization of the water. Therefore, the activity of water gets improved. Hence, hydration can improve concrete strength more efficiently [6]. Figure 14a,b represents the EDAX profile of the NWC and MWC specimens tested after 28 days of curing. The element composition of calcium and magnesium is reduced with a magnetic field intensity of 9000 Gauss, as shown in Figure 14b.

The EDAX analysis of MWC shows that the major portion of silica (31.95%) and a minimal amount of calcium (1.40%) are present in MWC compared to the NWC. The lower content of Ca and Mg shows the higher removal of hardness scale through magnetization, and the magnetic field tends to weaken the hydrated ions in calcium carbonate solution. Coey and Cass [46,49] reveal that the MFTW improves the amount of aragonite in the carbonate deposit rate with a 99.9% probability. The strength development of the MWC is authenticated by the presence of compounds such as ${\mathrm{SiO}}_{2}$ (higher proportionality) and CaO (lower proportionality) as observed in EDAX quantitative analysis [50].

## 4. Mechanism

**Figure 15:**
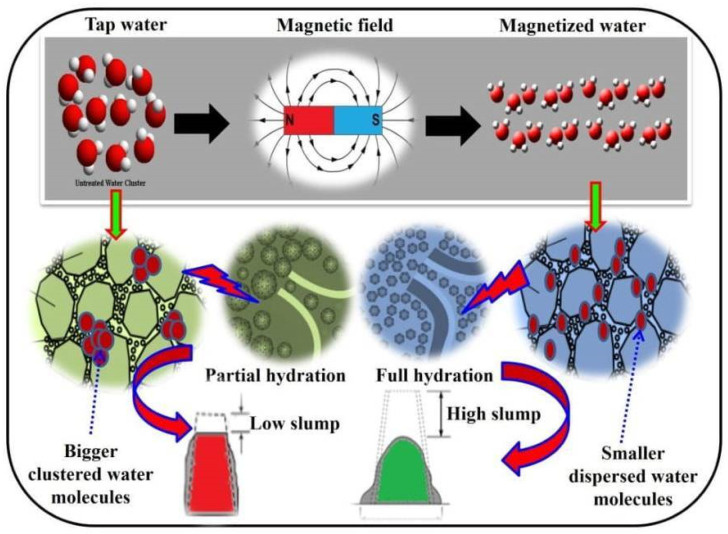
Mechanism of MFTW action on cement particles.

Moving fluids generate conductivity due to the magnetic field’s influence on water, leading to stresses on the fluids and changes in concentration. The physicochemical properties of the water molecules change due to the presence of kinetic energy in water particles up to some extent, which leads to the breakdown of water clusters into smaller ones. In normal water, water molecules are bigger in size and present in clustered form. When exposed to a magnetic field, the water molecules are disintegrated into discrete smaller molecules. The smaller distinct water molecules of magnetized water disperse uniformly in the concrete mix, reach each and every cement particle, and enhance the hydration process. Penetration of water molecules into the whole mass of concrete helps mobility of the binding materials and improves the workability of the concrete mix with a low water-cement ratio. In the concrete combination, the molecules have positive and negative parts. The positive part is loaded onto the cement particles and surrounds them. The cement particles’ electrical charges are turned negative by the magnetic field. This causes the cement particles to be separated and dispersed from one other, as shown in Figure 15. As a result, the MW molecules can easily penetrate the core region of cement particles to activate better hydration and improve the mechanical performance of concrete.

## 5. Conclusions

The experimental results show that the MFTW enhances the water parameters’ activation to improve the concrete’s strength when the MW is used for mixing. The following conclusions were reached:

- Magnetized water acts as a water reducing agent. Hence it can be very well used in the preparation of concrete mix without the addition of super plasticizer (SP) to improve the workability of concrete mix.
- Increase in the exposure duration of water to magnetic field improves the workability of concrete mix as compared to the workability of NWC mix with 1% SP.
- The slump value of concrete mix prepared with magnetized water obtained from instant exposure to a magnetic field and without the addition of super plasticizer is 96 mm whereas the slump value of normal water concrete mix with 1% SP is 98 mm. This proves that magnetized water in concrete mix without the addition of SP helps to realise a slump value more or less equal to that of normal concrete mix with 1% SP.
- The slump value of the MWC with 1% SP is 37% greater than that of the NWC with 1% SP.
- Utilizing the MW within 20 min after magnetization can be recommended to achieve the desired workability without SP.
- The improvement in compressive strength and split tensile strength of magnetized water concrete specimens amounts to 12% and 8.9%, respectively, as compared to that of NWC with 1% SP specimens.
- Microstructure development shows that the amount of CSH is larger and its morphology becomes denser and less porous with MWC mix.
- When concrete consisting of MW without SP and 30% of cement volume is replaced using granite powder waste, this improves the strength properties of M40 grade concrete.
- The proposed approach helps improve water quality in the concrete industry due to the water magnetization, which directly enhances the quality and life span of the structures. Moreover, the proposed technology can be used to achieve a sustainable and eco-friendly building structure.

## Figures and Tables

**Table 1:** Physical properties of ordinary Portland cement.

Physical Property	Test Value	Requirement as per IS: 12269-1987 [27]
Fineness	236 m^2^/Kg	225 m^2^/Kg
Setting time (Initial)	32 min	30 min (minimum)
Setting time (Final)	337 min	600 min (maximum)
Specific gravity	3.15	3.2
Standard consistency	36%	40%

**Table 2:** Chemical properties of cement.

Chemical Composition	Test Value (%)
CaO	62.21
${\mathrm{SiO}}_{2}$	23.72
${\mathrm{Al}}_{2}O_{3}$	6.12
${\mathrm{Fe}}_{2}O_{3}$	3.27
MgO	1.59
${\mathrm{Na}}_{2}O$	0.12
$K_{2}O$	0.19
LOI	0.98
Free CaO	0.52

**Table 3:** Aggregates’ physical properties.

Test	Coarse Aggregate	Fine Aggregate
Modules of fineness	3.43	4.80
Absorption (%)	0.10	1.00
Specific gravity	2.90	2.69

**Table 4:** Values of water quality parameters of normal water and magnetized water.

Parameter	Magnetization (mg/L)		
	Before	After	Allowable Limit
pH	8.1	8.5	6.5–8.5
Total hardness	600	380	600
Sulphate	134	115	<200
Chlorides	1025	552	1000
Total dissolved solids	1900	1258	2000
Iron	0.1	0.1	<1

**Table 5:** Mix proportions of concrete samples for 1 m^3^.

Mix	Cement (kg)	Granite Powder Waste (kg)	Potable Water (L)	Fine Aggregate (kg)	Coarse Aggregate (kg)	SP (%)
NW	456.58	-	174	661.03	1235	0
	456.58	-	174	661.03	1235	0.2
	456.58	-	174	661.03	1235	0.4
	456.58	-	174	661.03	1235	0.6
	456.58	-	174	661.03	1235	0.8
	456.58	-	174	661.03	1235	1
MW	456.58	-	174	661.03	1235	0
	456.58	-	174	661.03	1235	0.2
	456.58	-	174	661.03	1235	0.4
	456.58	-	174	661.03	1235	0.6
	456.58	-	174	661.03	1235	0.8
	456.58	-	174	661.03	1235	1
MW + Granite Waste	456.58		174	661.03	1235	0
	433.76	22.82	174	661.03	1235	
	410.93	45.65	174	661.03	1235	
	388.1	68.48	174	661.03	1235	
	365.27	91.31	174	661.03	1235	
	342.44	114.14	174	661.03	1235	
	319.61	136.97	174	661.03	1235	

## Data Availability

Not applicable.
